# Awareness and Perception of Plant-Based Diets for the Treatment and Management of Type 2 Diabetes in a Community Education Clinic: A Pilot Study

**DOI:** 10.1155/2015/236234

**Published:** 2015-01-31

**Authors:** Vincent Lee, Taylor McKay, Chris I. Ardern

**Affiliations:** ^1^Diabetes Education Centre, Southlake Regional Health Centre, Newmarket, ON, Canada L3Y 2B1; ^2^Department of Human Health and Nutritional Science, University of Guelph, Guelph, ON, Canada N1G 2W1; ^3^School of Kinesiology and Health Science, York University, Toronto, ON, Canada M3J 1P3

## Abstract

*Objective*. To assess awareness, barriers, and promoters of plant-based diet use for management of type 2 diabetes (T2D) for the development of an appropriate educational program. *Design*. Cross-sectional study of patients and healthcare providers. *Setting*. Regional Diabetes Education Centre in ON, Canada. *Participants*. *n* = 98 patients attending the Diabetes Education Centre and *n* = 25 healthcare providers. *Variables Measures*. Patient questionnaires addressed demographics, health history, and eating patterns, as well as current knowledge, confidence levels, barriers to, promoters of, and interests in plant-based diets. Staff questionnaires addressed attitudes and current practice with respect to plant-based diets. *Analysis*. Mean values, frequency counts, and logistic regression (alpha = 0.05). *Results*. Few respondents (9%) currently followed a plant-based diet, but 66% indicated willingness to follow one for 3 weeks. Family eating preferences and meal planning skills were common barriers to diet change. 72% of healthcare providers reported knowledge of plant-based diets for diabetes management but low levels of practice. *Conclusions and Implications*. Patient awareness of the benefits of a plant-based diet for the management of diabetes remains suboptimal and may be influenced by perception of diabetes educators and clinicians. Given the reported willingness to try (but low current use of) plant-based diets, educational interventions targeting patient and provider level knowledge are warranted.

## 1. Introduction

Diabetes has become a global epidemic affecting an estimated 371 million people (in 2012), a number that is expected to reach 552 million by 2030 [1]. With healthcare costs approaching $490 billion for the treatment of diabetes [2], alternative (patient-centered) lifestyle management approaches and cost-effective dietary interventions such as plant-based diets are a focus of increasing attention [3].

Recent research has revealed that 58% of type 2 diabetes (T2DM) cases can be prevented or delayed through lifestyle changes such as increased physical activity, healthy eating, and weight loss [3]. Other large cohort studies have shown that the prevalence of T2DM is significantly lower amongst people following a range of plant-based diets [4–6] and that those with greater adherence to plant-based foods, such as a low-fat vegan diet, experience the greatest benefit. Tuso et al. (2013) define a plant-based diet as a regimen that encourages whole, plant-based foods and discourages meats, dairy products, and eggs as well as all refined and processed foods [7]. (The definition of other variants of plant-based diets is included in the questionnaire.) Various studies suggest that plant-based diets can be an effective Medical Nutrition Therapy (MNT) for the treatment and management of T2DM [8], specifically by improving body weight, cardiovascular risk factors, and insulin sensitivity [9–11] and reducing the need for diabetic medications [12–14]. Providing MNT to people with diabetes demonstrates effectiveness in reducing hospitalization and physician services by 9.5% and 23.5%, respectively, which, in turn, reduces healthcare costs in the long-term [15]. Studies show that a plant-based diet is as effective, if not more effective than an ADA-recommended diabetes diet at reducing certain clinical markers such as HbA1c levels [14]. With the growing body of evidence, the new 2013* Canadian Diabetes Association Clinical Practice Guidelines* (CDACPG 2013) recommend the use of plant-based diets for management of T2DM [16]. However, this dietary pattern is often perceived to be extreme and difficult to follow, and this perception may be influenced by the healthcare providers that diabetic patients encounter. Despite a strong understanding of the health benefits of a plant-based diet, healthcare providers commonly cite low patient interest and difficulties in facilitating patient adoption as reasons for not promoting plant-based diets.

In order to provide insight into the justification for (and development of) an effective and patient-focused education program, a survey of patients and clinicians was undertaken to assess the awareness, confidence, perceived barriers and promoters, and educational needs for using a plant-based diet in the prevention and management of T2DM.

## 2. Methods

### 2.1. Participants and Study Design

The Diabetes Education Centre (DEC) at Southlake Regional Health Centre (SRHC) provides assessment and therapeutic and self-management education for adults with type 1, type 2, gestational diabetes, and prediabetes in York Region, ON, Canada. With the mission of providing a broad-based education on the prevention and management of diabetes, the DEC has approximately 12,500 patient visits annually. This pilot study was approved by the Research Ethics Board prior to patient enrolment and study commencement. Participants from the DEC community were subsequently recruited for one of two surveys: a patient survey or a health professional survey. All patients visiting the clinic for an appointment during the survey period were approached in the waiting room and given the option to complete the patient survey. The patient survey ran from April 22, 2013, to June 5, 2013, and 100 individuals agreed to participate. Inclusion criteria for patient participation included being a patient of the DEC diagnosed with prediabetes, type 1 diabetes, or type 2 diabetes. Patients with gestational diabetes and type 1 diabetes on insulin pump therapy and nonpatients were excluded from the study. The final analytic sample included 98 individuals (prediabetes: *n* = 14; type 1 diabetes: *n* = 17; and T2DM: *n* = 62).

### 2.2. Patient Questionnaire

Survey data was derived from dichotomous (yes/no) and Likert-type scale closed-ended questions. Additional open-ended questions were used to acquire more specific demographics, health history, and behavioural information (e.g., height, weight, and opinions about diabetes education needs). Since a validated questionnaire in this particular topic was not available, questions were carefully designed to address the following areas: (1) present knowledge, (2) confidence level, (3) potential barriers/promoters, and (4) interests and needs for establishing a future education program.

### 2.3. Staff Questionnaire

Staff members of the diabetes team were also asked to provide responses to a brief questionnaire on their attitudes and practices regarding plant-based diets. The health professional survey was offered to all staff members working at the DEC and included registered nurses (RN), endocrinologists, and registered dietitians (RD). The survey ran from March 25, 2013, to April 12, 2013, and was completed by 25 staff members: 11 RN, 1 endocrinologist, and 13 RD.

### 2.4. Data Analysis

Mean values (*μ*) and frequency counts (*n*, %) were used to describe the demographics, health history, and dietary practices and perceptions of participants for continuous and categorical variables, respectively. After developing a character profile of participants (e.g., Body Mass Index (BMI), diabetes type, duration of diabetes, new versus continuing patient, weight management strategies, etc.), logistic regression was used to explore the relationship between clinical and education-related factors on the willingness of patients to change to a vegetarian diet. All analyses were conducted using SPSS (v 19), with significance set at alpha = 0.05.

## 3. Results

### 3.1. Patient Knowledge and Perceived Barriers to Uptake of a Vegetarian Diet

In general, study participants tended to be male (55%), over age 50 (71%), be overweight or obese (73%), have T2DM (68%), be diagnosed in the last 10 years (65%), and be returning patients (55%) (Table 1). The majority of patients (89%) had not heard of using a plant-based diet to treat or manage T2DM. Furthermore, only 8 (9%) participants reported adherence to a plant-based diet of any type, 3 of whom had followed the diet for less than 1 year. Given the appropriate support, 66% of nonvegetarians were willing to follow a trial plant-based diet for 3 weeks. Nonetheless, almost half of participants cited concerns regarding “family eating habits” (48%), a lack of “meal planning skills” (45%), and a “preference to eat meat” (45%) as primary deterrents to following a plant-based diet. Other factors such as “food cost” (22%), “ease of cooking” (19%), “time constraints” (19%), and “other” factors (6%) were also common (results not shown). Few respondents were confident in their ability to follow a vegetarian (vegan, pesco-, or lacto-ovo) diet, with 17–28% of participants indicating that they were “not confident at all” (results not shown).

**Figure 1:**
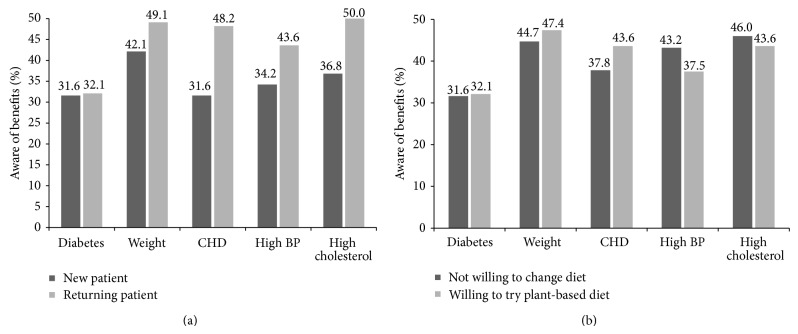
Percentage of patients who are aware of the benefits of a plant-based diet on various chronic conditions. Chi-square analysis comparing willingness to change diet and status of patient, all nonsignificant.

**Figure 2:**
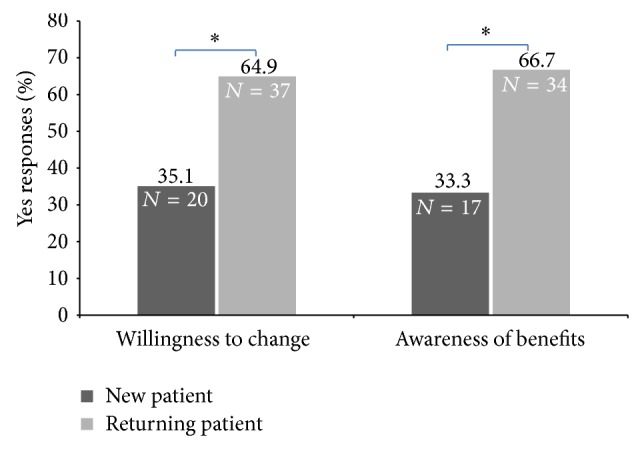
Awareness of the benefits and willingness to try a plant-based diet in new and returning patients. Chi-square analysis comparing new and returning patients; ^*^ *P* < 0.05.

Overall, less than half of all participants were aware of the benefits of a plant-based diet to improve diabetes, weight, heart disease, high blood pressure, or high cholesterol. Awareness also varied according to DEC attendance (Figure 1(a)) and willingness to try a plant-based diet (Figure 1(b)) While there was a trend for higher awareness within those who were willing to try plant-based diets and those who were returning patients in the DEC, these differences did not reach statistical significance (Figure 2).

When asked what supports would benefit dietary change, 22% of participants indicated that they did not intend on making a change. Stratified by time since diagnosis, more longer-term than newly diagnosed diabetics (30% versus 10%, *P* < 0.05) were unwilling to consider a plant-based diet, despite greater awareness of alternative dietary treatments to diabetes. At the bivariate level, patients interested in educational resources (OR = 42.9, 95% CI: 12.9–142.4) and those who were motivated by potential health (13.0, 4.9–34.2) or weight loss (4.0, 1.6–9.6) benefits of a plant-based diet had higher odds of being willing to make the necessary change (Table 2). Further adjustment for age and sex only served to strengthen the association between motivation and willingness to change [health: 17.0 (5.9–48.8); weight loss: 4.8 (1.9–12.0)].

### 3.2. Staff Perception and Use of Plant-Based Diets

A majority of staff (72%) were aware of the use of plant-based diets for treatment of T2DM, but only 32% are currently recommending this dietary pattern to patients (Table 3). While the reasons are likely to vary by clinician and individual patient risk profile, the three most commonly cited reasons were as follows: (1) this eating pattern is not realistic and too difficult to adhere to (and could lead to meal imbalance); (2) there is low perceived acceptance by patients; (3) there are lack of clear clinical practice guidelines and diet-specific educational support.

## 4. Discussion and Conclusion

### 4.1. Discussion of Patient Questionnaire Results

Study results reflect that approximately 89% of patients were not aware of using an alternate diet such as a plant-based diet for the prevention and management of T2DM and many of them cited low confidence in adopting this eating pattern. However, two-thirds of the patients showed willingness to follow a plant-based diet for the short-term and expressed interest in attending a vegetarian education program. Patients' low awareness and confidence level on the use of plant-based diets for managing T2DM can be partially attributed to the fact that, despite the growing interest in the health benefits of a plant-based diet, the vegetarian population remains relatively small in Canada (4%) [17]. Another plausible reason is that the patients were not well informed about this eating pattern, as less than half of the diabetes team recommended this dietary pattern to patients, potentially influencing their awareness and confidence level.

The top three barriers for making dietary changes towards a plant-based diet included family's influence, preference of eating meat, and meal planning skills. To promote this change, patients cited their top educational needs to be a vegetarian education program consisting of individual or group counselling and cooking instructions components. This result suggests that the traditional theory-based nutrition education at the DEC setting may be insufficient to address patients' barriers; a bigger focus should be placed on the practical aspects, such as teaching patients and family members how to prepare appetizing plant-based meals, in order to change their perception towards this new eating pattern.

### 4.2. Discussion of Staff Perception

One of the common reasons for diabetes educators not to be recommending this diet to patients was that this dietary approach is too difficult to follow with low perceived approval (i.e., patients are unlikely to accept it). This notion is contrary to the patient survey results that almost two-thirds of patients were willing to follow this dietary pattern at least for short-term when educational support is provided. Katcher et al. (2010) also indicate in a workplace study that a vegan diet is well accepted with over 95% adherence rate, and subjects report increased energy level, better digestion, better sleep, and increased satisfaction when compared with the control group [18]. Participants recruited for the Katcher et al. study that had a BMI ≥ 25 and/or previous diagnosis with type 2 diabetes were randomized into a low fat vegan diet group or a placebo group for 22 weeks. The treatment group received weekly instructions but no meals were provided [18]. Previous studies also demonstrate that the adherence and acceptability of a vegetarian diet are comparable to those of other therapeutic diets [19–21]. A number of staff members also expressed their second reason for not recommending this diet as the clinical practice guidelines and scientific evidence regarding this dietary pattern are not clear. Although randomized controlled intervention studies regarding the use of plant-based diets for the treatment of diabetes have been rather limited until recently, a number of high profile studies show that this diet is not only nutritionally adequate for long-term use [14, 22] but also effective in promoting weight loss, reducing insulin resistance, reducing diabetes medications (43% versus 5%) [10], and improving plasma lipids levels and overall glycemic control [12, 13]. Studies show that a plant-based vegan diet may be as effective as an ADA-recommended diet at causing weight loss [12, 13] and decreasing fat intake [22]. It may be more effective than an ADA-recommended diet at reducing the use of diabetes medication, HbA1c levels, and plasma lipids [13]. It is therefore possible that our survey simply captured a lag-time in dissemination of this new information from current research findings to clinician to patient. Alternatively, because there are a number of vegetarian food guides and practical guidelines that can be used by nutritional professionals [14, 23, 24], the disparity in recommendations and the varying effect of plant-based diets may in themselves be a challenge for nutrition counselling.

### 4.3. Limitations

As with any study, the results of this preliminary survey must be interpreted with caution. First, the small sample size (*n* = 98) and selective nature of participant recruitment limit the applicability of the results to a larger and more general population. Second, the patients who completed the surveys may be individuals who were already more interested in vegetarian diets and have a healthier risk profile overall. It is also possible that not all patients understood the terminology being used. When designing the patient and staff questionnaires, it was intended to compare the acceptance and confidence levels of using different types of vegetarian diets such as lacto-ovo vegetarian, pesco-vegetarian, semivegetarian, and vegan diets. However, as many patients had limited exposure to these diets, many of these questions were left unanswered or a single response was selected as an overall rating for all diet types. The questions used in both surveys were not validated due to the preliminary nature of the study as well as a lack of validated questionnaires available for use in this particular subject area. This may limit the ability to compare the current study to other similar studies. Although more than half (66%) of patients expressed an interest in following a plant-based diet, this interest was expressed only for the three-week period; therefore it does not guarantee long-term interest in or attendance in education programs or the longer-term change in eating habits that would be required for effective diabetes management. Finally, the heterogeneity of the surveyed clinicians limits the ability of the current study to ascertain a certain opinion of a given professional group.

### 4.4. Conclusion

Patient awareness of (and interest in) the benefits of a plant-based diet for the management of diabetes remains suboptimal and may be influenced by the perception of diabetes educators and clinicians. To provide assurance of the acceptability and efficacy of plant-based diets to patients, offering diet-specific education programs by nutrition professionals in community-based diabetes centres is warranted. Developing these programs in partnership with local nutrition service providers such as community kitchens, grocery stores, and local food network could foster exchange of teaching experience and new perspectives amongst educators and enable sharing of important teaching resources such as a demonstration kitchen. As such, additional training on plant-based diets may require the development of a more standardized and user-friendly practice guideline on plant-based diets to facilitate patient education. With its proven multiple health benefits, a plant-based diet has clearly shown to be beneficial in improving clinical outcomes, and also it has great potential to alleviate healthcare cost in the prevention and management of diabetes as well as other chronic diseases. The current study provides support for the need to further investigate the cost-effectiveness of this dietary pattern in a clinical setting.

### 4.5. Implications for Future Practice

There is now considerable evidence to support the use of plant-based diets as an effective Medical Nutrition Therapy for chronic diseases such as T2DM [8, 10, 12–14]. Nonetheless, results from this pilot study suggest low awareness and confidence, but a willingness to try a plant-based eating pattern, which supports the need for a patient-focused vegetarian education program. For diabetes educators and registered dietitians, developing diet-specific education programs (such as the Mediterranean and vegetarian diets) are now supported within best practice guidelines such as CDACPG 2013. Depending on available resources, the nutrition program should consist of one-on-one or group counseling sessions and cooking instructions components, and educators should address the patient's barriers to change (e.g., family's eating preference and meal planning skills) to increase a patient's likelihood of making long-term lifestyle changes.

## Figures and Tables

**Table 1:** Knowledge and perception of plant-based diets in patients attending a Diabetes Education Centre.

Age	
<50 y	28 (28.6%)
≥50 y	70 (71.4%)
Sex (% male)	54 (55.1%)
Body Mass Index	
Normal weight (18.5–24.9 kg/m^2^)	26 (26.5%)
Overweight (25.0–29.9 kg/m^2^)	26 (26.5%)
Obese (≥30.0 kg/m^2^)	46 (46.9%)
Diabetes type	
Prediabetes	14 (15.1%)
Type 1 diabetes	17 (18.7%)
Type 2 diabetes	62 (68.1%)
Time since diagnosis of diabetes^*^	
0–10 years	57 (65.5%)
10+ years	30 (34.5%)
Dietary practices	
Not on plant-based diet	85 (91.4%)
Semivegetarian	6 (6.5%)
Pesco-vegetarian	2 (2.2%)
Patient history in diabetes clinic	
New patient	41 (44.6%)
Returning patient	52 (55.4%)

Note: values may not add up to 100% due to missing responses and rounding.

^*^excludes *N* = 11 prediabetics.

Values for continuous measures are *µ*. Categorical measures are *N* (%).

**Table 2:** Unadjusted logistic regression between clinical and patient-education factors on willingness to change to a vegetarian diet^*^.

	Odds ratio (95% CI)
Patient interest in education on vegetarian diets	
No	1.0 (referent)
Yes	**42.9** (**12.9–142.4**)
Interest in plant-based diet is to improve health	
No	1.0 (referent)
Yes	**13.0** (**4.9–34.2**)
Interest in plant-based diet is to lose weight	
No	1.0 (referent)
Yes	**4.0** (**1.6–9.6**)
Age	
18–29 y	1.0 (referent)
30–49 y	4.0 (0.6–27.4)
50–65 y	3.5 (0.6–20.1)
65+ y	1.60 (0.2–11.1)
Sex	
Female	1.0 (referent)
Male	1.3 (0.6–2.9)
Demographic and clinical characteristics	
Prediabetes	1.0 (referent)
Type 1 diabetes	1.4 (0.3–5.9)
Type 2 diabetes	1.0 (0.3–3.1)
Time since diabetes diagnosis	
0–10 y	1.0 (referent)
10+ y	1.3 (0.5–3.1)
Body Mass Index	
Normal weight (18.5–24.9 kg/m^2^)	1.0 (referent)
Overweight (25.0–29.9 kg/m^2^)	0.9 (0.3–2.6)
Obese (≥30.0 kg/m^2^)	1.1 (0.4–3.0)
Confidence in becoming vegetarian	
Somewhat confident or confident	1.0 (referent)
Not at all confident	1.2 (0.5–2.9)
Heard of a plant-based diet	
No	1.0 (referent)
Yes	2.1 (0.5–8.3)
Aware of benefits of a plant-based diet	
No	1.0 (referent)
Yes	1.3 (0.6–2.8)
Patient history in diabetes clinic	
First visit	1.0 (referent)
Returning patient	1.3 (0.5–3.1)

^*^Sample includes only participants who are not currently on a plant-based diet (*N* = 85). Significant associations are presented in bold.

**Table 3:** Staff perception and recommendation for patient use of plant-based diets.

Heard of using a plant-based diet to treat diabetes	
Yes	18 (72.0%)
No	6 (24.0%)
No response	1 (4.0%)
Perceived confidence planning a plant-based diet	
Confident	8 (32.0%)
Somewhat confident	3 (12.0%)
Not confident	10 (40.0%)
No response	4 (16.0%)
Current practice regarding plant-based diets	
Currently recommending	8 (32.0%)
Not recommending	14 (56.0%)
No response	3 (12.0%)

Note: values may not add up to 100% due to missing responses and rounding.

Numbers are *N* (%).
